# ﻿Comparative morphology of part of the integumental fine structure of two Erythroneurine species: *Singaporashinshana* (Matsumura, 1932) and *Empoascanarasipra* Dworakowska, 1980 (Hemiptera, Cicadellidae, Typhlocybinae)

**DOI:** 10.3897/zookeys.1103.80787

**Published:** 2022-05-26

**Authors:** Jia Jiang, Christopher H. Dietrich, Can Li, Yuehua Song

**Affiliations:** 1 School of Karst Science, Guizhou Normal University / State Engineering Technology Institute for Karst Desertification Control, Guizhou, Guiyang, 550001, China Guizhou Normal University Guiyang China; 2 Illinois Natural History Survey, Prairie Research Institute, University of Illinois, 1816 S. Oak St., Champaign, IL 61820, USA University of Illinois Champaign United States of America; 3 Guizhou Provincial Key Laboratory for Rare Animal and Economic Insect of the Mountainous Region, Guiyang University, Guiyang, Guizhou, 550005, China Guiyang University Guiyang China

**Keywords:** Antennae, brochosomes, fine structure, forewings, karst, mouthparts

## Abstract

This study describes the fine structure of the mouthparts, antennae, forewings, and brochosomes of two leafhopper species belonging to the typhlocybine tribe Erythroneurini collected from the Karst area of Guizhou Province, southern China: *Singaporashinshana*, which prefers woody dicot hosts, and *Empoascanarasipra*, which feeds on grasses. As in other leafhoppers, the piercing-sucking mouthparts consist of a conical labrum, a cylindrical three-segmented labium, and a slender stylet fascicle. The labrum of both species has no sensilla and the labium has several common types of sensilla, but the two species differ in the numbers, types, and distribution of sensilla and in other aspects of the surface sculpture of the mouthparts. The stylet fascicle has distinctive dentition on both the maxillary and mandibular stylets. The antennae of the two species differ in several respects, including the sensilla and sculpture of the scape, pedicel, and flagellum, as well as the degree of sub-segmentation of the flagellum. Except for the variable scaly structure and rounded protrusions on the surface of *S.shinshana*, the fine structure of the forewing surfaces of the two species are similar to those of other leafhoppers. Only small spherical brochosomes were found on the body surface of *S.shinshana* and *E.sipra*. Similar studies of additional erythroneurine species are needed to determine whether differences in mouthpart and antennal fine structure may reflect adaptation to different host plant.

## ﻿Introduction

Leafhoppers, the Cicadellidae, are the largest family of Hemiptera and are widely distributed in six zoogeographic regions with more than 2,600 genera and 22,000 species (Oman et al. 1990; Dietrich 2005). Leafhopper nymphs and adults use piercing-sucking mouthparts to pierce the surface of the plant and suck either the phloem or xylem sap, or leaf parenchyma cell contents. The latter type of feeding is restricted to the subfamily Typhlocybinae and causes characteristic white spots on the leaves, which may cause the leaves to wither and fall off (Backus and McLean 1982; Leopold et al. 2003). Some leafhoppers are vectors of viral or bacterial plant pathogens, which can cause plant diseases, such as the common maize chlorotic dwarf virus, rice waika virus, and the recently discovered wheat yellow striate virus (Hirao and Inoue 1979; Hunt and Nault 1990; Yan et al. 2018). The feeding strategies of leafhoppers and their potential for rapid reproduction, often make them difficult to control using conventional pest management strategies and their impacts on yield and quality of crops may be severe.

Over the course of their more than 400 million years of evolution, different insects have acquired a wide variety of integumental structures, including sensilla and sculpturing that enabled them to interact and adapt to various environmental conditions. Such structures play important roles in finding hosts, mating, and defense. Using light microscopy and scanning electron microscopy, Willis (1949), Moulins (1971), Rice (1973), and others successively studied the fine structure on body surfaces of insect and characterized various sensilla found on different body parts and regions. Within the order Hemiptera, the fine structure of aphids has been studied extensively, especially their feeding structure (Davidson 1914; Forbes 1977; Pointeau et al. 2012). Another economically important group of Hemiptera, the leafhoppers, also have a large variety of sensilla and epidermal structures, but their morphology, types, and quantity are quite different from those of other hemipterans (Backus and McLean 1982; Brozek et al. 2006; Zhang et al. 2016). Leafhoppers appear to be unique among insects in producing brochosomes, tiny proteinaceous particles produced in the Malpighian tubules and spread over the body as a hydrophobic coating (Rakitov and Carolina 2005; Rakitov 2009). Brochosomes are often deposited on a particular area of the forewing called the brochosome field prior to being spread over the rest of the body. The mouthparts of leafhoppers are very similar overall to those of other Hemiptera in having a modified, elongated labrum, labium, and stylet fascicle, but their shape, segmentation and fine structure differ from those of other hemipterans (Tavella and Arzone 1993; Hao et al. 2016a; Ge et al. 2016). Leafhoppers have three-segmented antennae, and the structural variation appears to be relatively low compared to other Hemiptera (Mazzoni et al. 2009; Zhang et al. 2016). However, relatively few studies so far have focused on the fine structure of leafhoppers, and these mostly focused on representatives of a single subfamily, Deltocephalinae, that includes vectors of various plant pathogens (Backus and McLean 1982; Zhao et al. 2010; Liu et al. 2020; Zhang et al. 2020). Such studies have not been performed on Typhlocybinae, which mostly includes species that feed on leaf parenchyma cell contents and, therefore, occupy a different feeding niche from other leafhoppers.

To date, the fine structure of the integument of Typhlocybinae remains largely unstudied. Dong and Huang (2013) described the anointing behavior of *Singaporashinshana* (Matsuma, 1932)and mentioned the morphology of the brochosomes but did not study or illustrate the fine structure of brochosomes and other features of the integumental fine structure. This paper provides the first detailed SEM study of the integumental fine structure of species of Typhlocybinae, focusing on the mouthparts, antennae, forewings, and brochosomes of two species of Chinese Erythroneurini.

## ﻿Materials and methods

**Figure 1.**
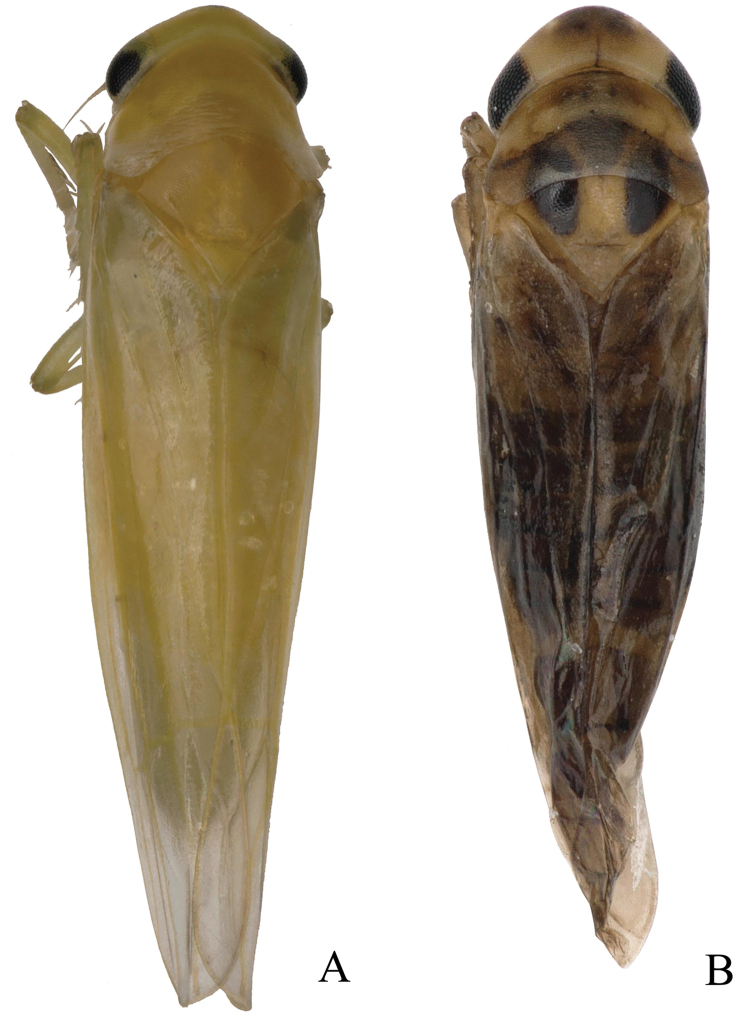
**A** habitus of *Singaporashinshana*, dorsal view **B** habitus of *Empoascanarasipra*, dorsal view.

The adult specimens of *S.shinshana* were collected on a peach tree on the Baoshan Campus of Guizhou Normal University, Guiyang City, Guizhou Province, China (26°35'30"N, 106°43'9"E) on 21 June 2020. The temperature at the time of collection was 27 °C, and the humidity was 91%. The adult specimens of *E.sipra* Dworakowska, 1980 were collected on *Festucaelata* Keng ex E. Alexeev, 1977 in Changpoling Forest Park, Guiyang City, Guizhou Province, China (26°38'45"N, 106°39'10"E) on 27 June 2020. The temperature was 20 °C and the humidity was 99% during collection. The overall appearance of the two leafhopper species is shown in Fig. 1. All specimens examined are deposited in the collection of the School of Karst Science, Guizhou Normal University, China (**GZNU**).

Newly captured adult specimens were placed in a -24 °C freezer for 20 min. Then ten frozen specimens (5 males and 5 females) were selected at random and dissected under a stereo microscope (Olympus SZX16, Japan), with the head and wings removed on dry filter paper, then placed in 2.5% glutaraldehyde fixative at 4 °C for 12 hours. Specimens were subsequently transferred to phosphate buffer saline (PBS, 0.1M, pH7.2) and rinsed five times, 5 min each time. Dissected parts (except wings) were then placed in an ultrasonic cleaner for 30 s, and then dehydrated in a graded series of 30%, 50%, 70%, 90%, 95%, and 100% acetonitrile for 20 min. Thereafter, the samples were mounted on aluminum stubs with double-sided sticky copper tape and sputtered with gold/palladium in a JEOL JFC-1600 high resolution sputter coater. The samples were subsequently examined with a JSM-6490LV SEM operated at 20 kV. The measurement data were obtained by scanning electron microscope.

General terminology for the classification of sensilla follows Altner and Prillinger (1980) and Zacharuk (1980) with terminology more specific to leafhopper structures following more recent authors (Rakitov 2000; Zhao et al. 2010; Stacconi and Romani 2012; Brozek and Bourgoin 2013; Ge et al. 2016; Hao et al. 2016a, b). Sensilla classification is summarized in Table 1.

**Table 1.** Classification of sensilla and cuticular processes.

**Type**		**Features**	**Reference images**
Sensilla trichodea	S.t. I	Hair-like, slender, slightly curved, length ≥ 20 μm.	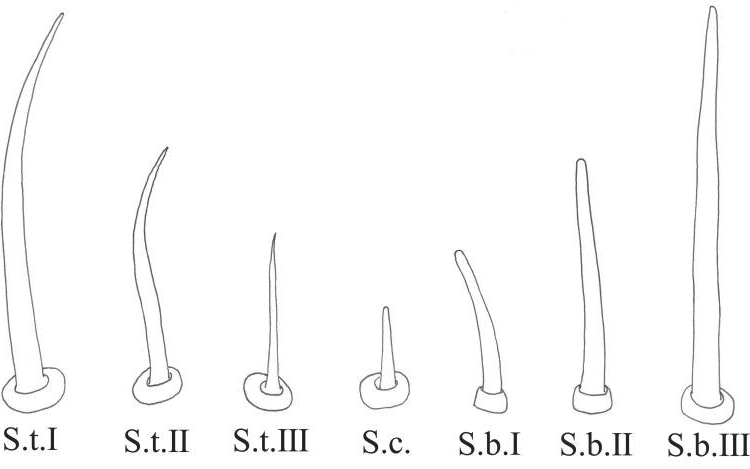
	S.t. II	Relatively short.	
	S.t. III	Short and thin, length ≤ 10 μm.	
Sensilla chaetica	S.c.	Shaped like short spines, erect or curved along the axis.	
sensilla basiconica	S.b. I	Upright or curved along the axis, the top is blunt, thick and short, length ≤ 10 μm.	
	S.b. II	Relatively thick and long.	
	S.b. III	Thick and long, length ≥ 20 μm.	
Peg sensilla	Pg.s.u. I	Peg-like, length 2.0~5.0 μm.	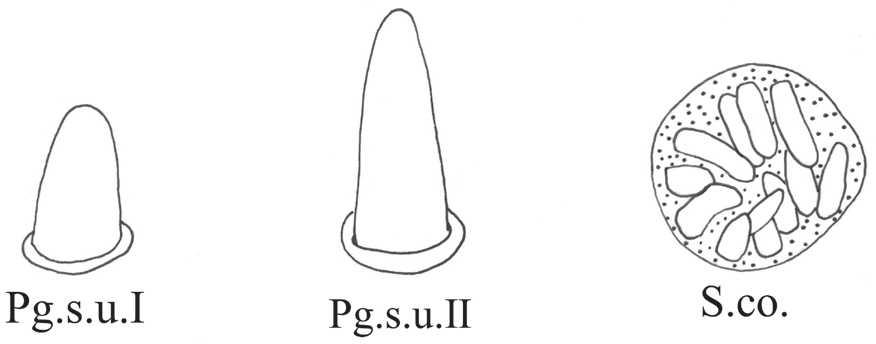
	Pg.s.u. II	Peg-like, length 5.0~7.0 μm.	
Sensilla coeloconica	S.co.	A cluster of finger-like structures arranged in a round concavity, 6–16 finger-like protrusions.	
Scaly structures	Sc.s.	A scaly protrusion or a scaly structure composed of many small protrusions (non-sensilla).	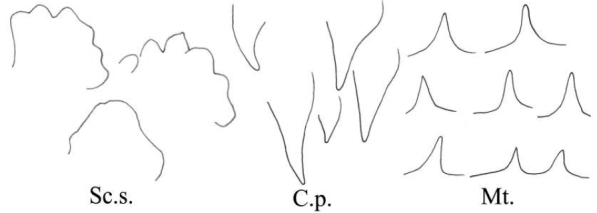
Cuticular processes	C.p.	Triangular protrusions with thin and pointed ends (non-sensilla).	
Microtrichia	Mt.	Small rigid projections occurring singly or in groups of two or three arranged together (non-sensilla).	

## ﻿Results

**Figure 2.**
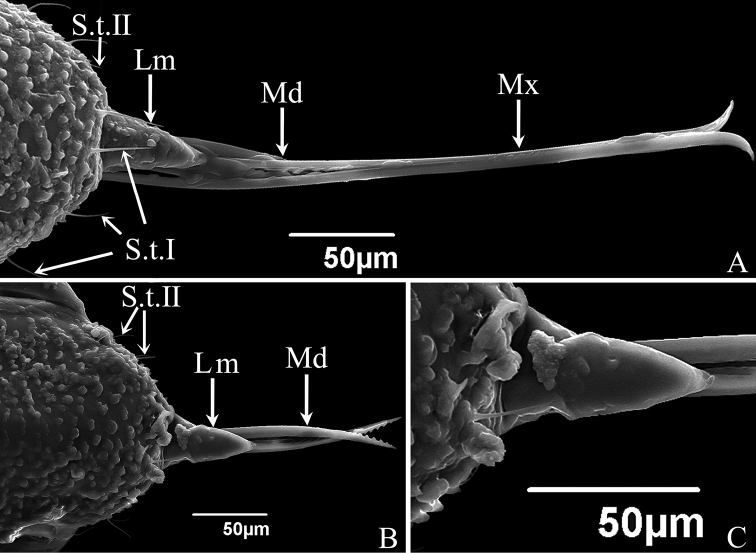
SEM of the mouthparts of *S.shinshana***A** anterior view, showing labrum (Lm), mandibular stylets (Md), maxillary stylets (Mx), sensilla trichodea I (S.t. I) and sensilla trichodea II (S.t. II) **B** anterior view of anteclypeus and labrum (Lm), showing irregular protrusions on surface of anteclypeus, labrum, mandibular stylets (Md) and sensilla trichodea II (S.t. II) **C** cone-shaped labrum showing a smooth surface.

**Figure 3.**
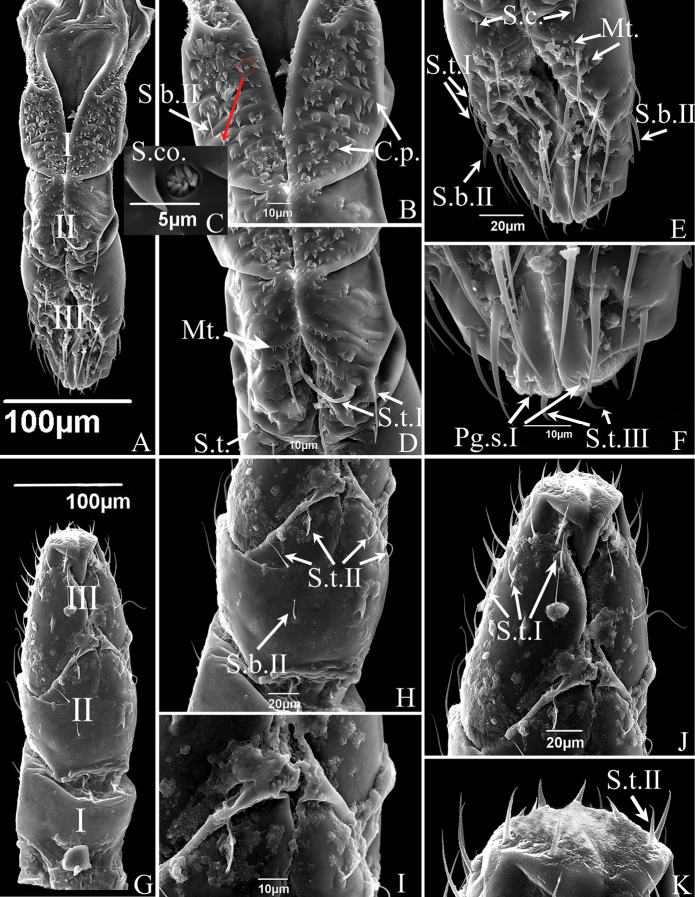
SEM of the labium of *S.shinshana***A** anterior view of labium showing three-segmented labium (I-III), and sensilla symmetrically located on each side of the labial groove **B** anterior view of first segment of labium showing sensilla basiconica II (S.b.II) and cuticular processes (C.p) **C** sensilla coeloconica (S.co.) **D** the anterior view of second segment of labium showing sensilla trichodea I (S.t.I) and microtrichia (Mt.) **E** anterior view of third segment of labium showing sensilla trichodea I (S.t.I), sensilla basiconica II (S.b.II), sensilla chaetica (S.c.) and microtrichia (Mt.) **F** anterior view of labial tip showing peg sensilla I (Pg.s.I) and sensilla trichodea III (S.t.III) **G** dorsal view of mouthparts showing three-segmented labium (I-III) and some sensilla **H** dorsal view of second segment of labium showing sensilla trichodea II (S.t.II) and sensilla basiconica II (S.b.II) **I** junction of second and third labial segments showing spherical protrusions **J** dorsal view of third segment of labium showing sensilla trichodea I (S.t.I) **K** tip of labium, showing distribution of sensilla.

The mouthparts of *S.shinshana* and *E.sipra* are typical piercing-sucking mouthparts, consisting of a labrum (Lm), labium (Lb), two mandibular stylets (Md), and two maxillary stylets (Mx) comprising the stylet fascicle (Sf) (Figs 2, 5). The three-segmented labium has a deep longitudinal groove (Lg) on the anterior surface that houses and protects the stylet fascicle (Figs 3A, 5A). Except for the difference in size, the shape of the mouthparts and the distribution of sensilla are not different between male and female adults. Measurements are summarized in Table 2. The distribution and numbers of sensilla are summarized in Table 4.

**Table 2.** Measurements of labrum and labium (mean ± SE) obtained from scanning electron microscopy, *n* = 5. Lm: labrum; Lb: labium; Lb-1: first segment of labium; Lb-2: second segment of labium; Lb-3: third segment of labium.

Segment		Lm		Lb-1		Lb-2		Lb-3		Lb total length	
		*S.shinshana*	*E.sipra*	*S.shinshana*	*E.sipra*	*S.shinshana*	*E.sipra*	*S.shinshana*	*E.sipra*	*S.shinshana*	*E.sipra*
Length (μm)	Male	62.7±12.0	52.37±3.2	81.3±8.7	73.1±7.9	90.8±10.3	73.7±5.6	108.3±5.4	96.0±15.1	280.4±24.4	242.8±28.6
	Female	72.7±9.8	69.4±10.1	96.1±16.1	78.6±5.2	99.7±9.2	84.6±3.5	122.1±6.4	114.4±7.9	317.9±31.7	275.6±16.6

The labrum is conical in shape and connected to the apical margin of the anteclypeus. The anteclypeus has many irregular protrusions on its surface, with some sensilla trichodea I and sensilla trichodea II symmetrically distributed on its surface (Figs 2, 5D). The labrum has a smooth surface, except for a few slight bumps (Figs 2, 5D).

The labium consists of three cylindrical segments (Figs 3A, G, 5A); its length varies in proportion to the overall body size of individuals. The length relationship between the three labial segments is: I < II < III; the first and second segments are almost equal in length, the third segment is distinctly longer. The first labial segment is smooth on the surface without sensilla in dorsal view (Figs 3G, 5G). The anterior surface in *S.shinshana* has two sensilla coeloconica (~ 2.64 μm in diameter) and a sensilla basiconica II. Sensilla basiconica I are symmetrically distributed, sensilla basiconica II is distributed only at one side, a very rare occurrence(Fig. 3B, C). *Empoascanarasipra* has two sensilla basiconica I and two sensilla trichodea II symmetrically distributed in anterior view (Fig. 5K). Numerous transverse wrinkles are present on the anterior surface of the first labial segment of *S.shinshana*, and many small spinelike cuticular processes < 8 μm in length are clearly visible (Fig. 3B). These cuticular processes all have the same distal orientation, and are scattered on the anterior surface of the first and second labial segments, but the second segment only has a few cuticular processes near the junction with the first segment (Fig. 3B, D). The first labial segment of *E.sipra* also has many transverse wrinkles but differs from *S.shinshana* in having groups of small microtrichia instead of larger spinelike processes (Fig. 5K).

A few microtrichia are concentrated on oblique ridges near the median longitudinal groove on the second labial segment of *S.shinshana*, while a larger number of microtrichia are distributed on the second and third labial segments of *E.sipra* (Figs 3D, E, 5H–M). Twelve sensilla trichodea I are distributed asymmetrically on both sides of the groove of *S.shinshana.* Four sensilla trichodea II are symmetrically distributed on the dorsal surface of the second labial segment, and one sensilla basiconica II is present on the left side (Fig. 3H, the sensillum on the opposite side may have fallen off). The junction of the second and third labial segments in dorsal view is heavily sclerotized, forming a raised ridge, and a round protrusion is present in middle of the ridge (Fig. 3I). The second labial segment of *E.sipra* has six sensilla trichodea II, which are symmetrically distributed on both sides of the groove, and eight sensilla trichodea I are symmetrically distributed on the second labial section and close to the third labial segment in anterior view; two sensilla trichodea I and four sensilla trichodea II are symmetrically distributed in dorsal view (Fig. 5H, L).

The third labial segment is longer than other two segments, gradually tapered towards the apex, and more densely covered with sensilla, mostly symmetrically distributed. Sensilla trichodea I–III, peg sensilla (*S.shinshana*: peg sensilla I, ~ 3.32 μm in length; *E.sipra*: peg sensilla II, ~ 5.57 μm in length) and sensilla basiconica (*S.shinshana*: sensilla basiconica II, ~ 18.05 μm in length; *E.sipra*: sensilla basiconica I, ~ 9.58 μm in length) are distributed on the third labial segment of the two species; and there is a pair of peg sensilla distributed on both sides of the longitudinal groove. The labial tip surface is uneven, with many small, rounded protrusions (Figs 3E, F, J, K, M, 5I, J,). The majority of sensilla trichodea of *E.sipra* are arranged in an obvious order, while the sensilla trichodea of *S.shinshana* are scattered (Figs 3E, 5M). In addition, two sensilla chaetica are distributed on the right side of the third segment of *E.sipra*, adjacent to the second segment (Fig. 5M).

**Figure 4.**
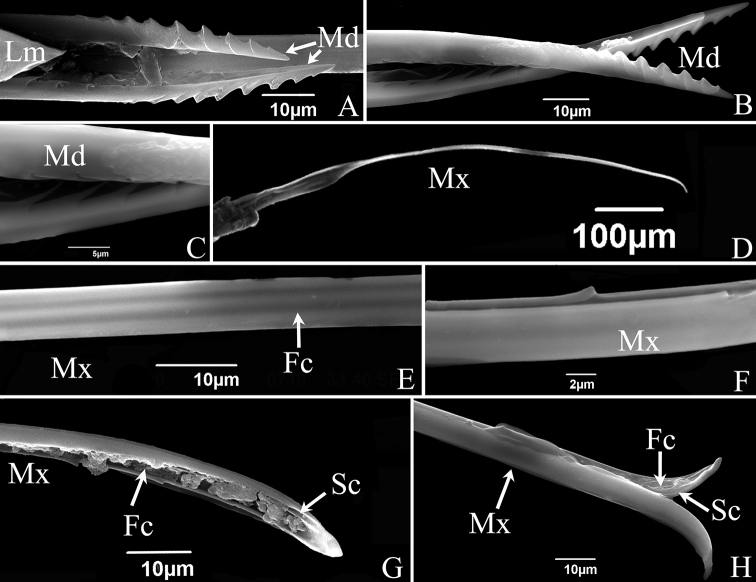
SEM of the stylet fascicle of *S.shinshana***A** mandibular stylets (Md), showing relative position of mandibular stylets and labrum (Lm) **B** mandibular stylet (Md), showing serrate ridge on the convex external surface and zigzag structure on inner edge **C** enlarged middle of mandibular stylet (Md), showing zigzag structure on inner edge **D** maxillary stylets **E** dorsal view of middle section of maxillary stylets (Mx), showing lines indicating food canal (Fc) **F** lateral view of middle section of maxillary stylets (Mx), showing relatively blunt tooth-like protrusion **G** tip of maxillary stylet (Mx), showing salivary canal (Sc) and food canal (Fc) **H** tip of maxillary stylets (Mx), showing two stylets with different lengths.

**Figure 5.**
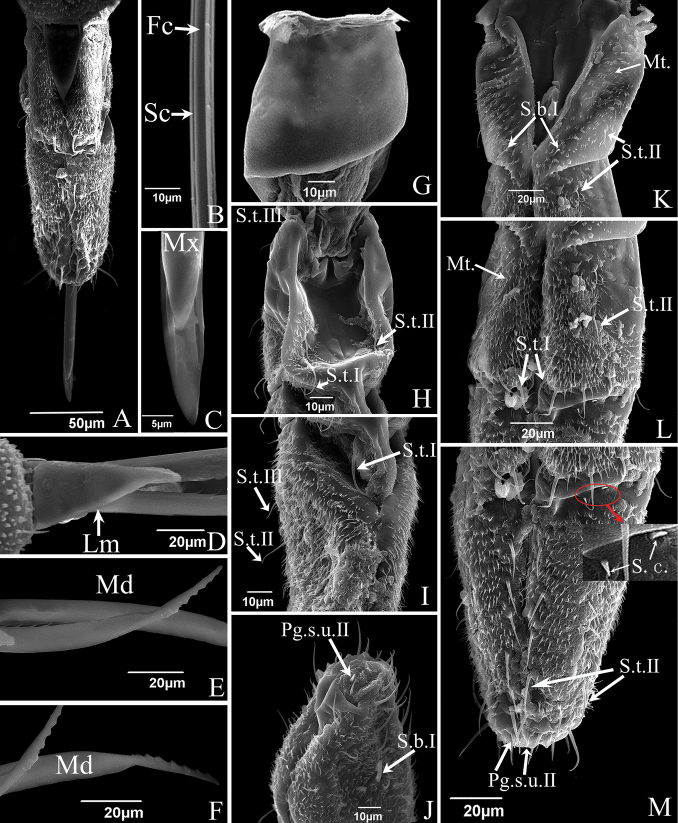
SEM of the mouthparts of *E.sipra***A** the anterior view of labrum and labium showing sensilla symmetrically located on each side of the labial groove or around the tip of the labium **B** one of the maxillary stylets (Mx) showing food canal (Fc) and salivary canal (Sc) **C** the enlarged view of the tip of maxillary stylets (Mx) which are pointed and incurred **D** cone-shaped labrum showing a smooth surface **E** mandibular stylet (Md), showing serrate ridge on the convex external surface and zigzag structure on inner edge **F** mandibular stylet (Md), showing the depression on the side of the mandibular stylet **G** dorsal view of first segment of labium showing a smooth surface **H** dorsal view of second segment of labium showing sensilla trichodea I (S.t.I) and sensilla trichodea II (S.t.II) **I** dorsal view of third segment of labium showing sensilla trichodea I (S.t.I), sensilla trichodea II (S.t.II), sensilla trichodea III (S.t.III) **J** tip of labium, showing sensilla basiconica I (S.b.I) and peg sensilla II (Pg.s.II) **K** anterior view of first segment of labium showing sensilla basiconica, II (S.b.II) and cuticular processes (C.p) **L** anterior view of second segment of labium showing sensilla trichodea I (S.t.I), sensilla trichodea II (S.t.II) and microtrichia (Mt.) **M** anterior view of third segment of labium showing sensilla trichodea II (S.t.II), sensilla chaetica (S.c.) and peg sensilla II (Pg.s.II).

The stylet fascicle is composed of paired, elongated mandibular and interlocking maxillary stylets. The mandibular stylets partially sheathe the maxillary stylets laterally and are significantly shorter than the latter. They are crescent-shaped in cross-section, thus forming a deep groove enclosing the maxillary stylets. Each mandibular stylet has a row of slender tooth-like protrusions on its inner edge in the basal half, and the protrusions together form a zigzag structure (Figs 4B, C, 5E); the outer surfaces of the distal half have a serrate ridge consisting of eight or nine, more or less evenly spaced, teeth (Figs 4A, B, 5E, F). The mandibular stylets of *S.shinshana* have a wider base that gradually narrows toward the apex; the mandibular stylets of *E.sipra* suddenly narrow at the base of the serrate ridge, then slightly expand, and then gradually narrow toward the apex (Figs 4A, B, 5E, F).

The two maxillary stylets are semicircular in cross-section and tightly interlocked to form a salivary canal (Sc) and a food canal (Fc) (Figs 4G, H, 5B, C). The maxillary stylets are elongated, smooth on the outer surface, but longitudinal lines representing the food canal can be clearly seen (Fig. 4D–F); widely spaced, blunt, tooth-like protrusions are present on the two sides and prevent them from separating during feeding (Figs 4F, 5B). The two maxillary stylets are asymmetrical and differ in length; with sharp tips used to pierce plant tissues.

**Figure 6.**
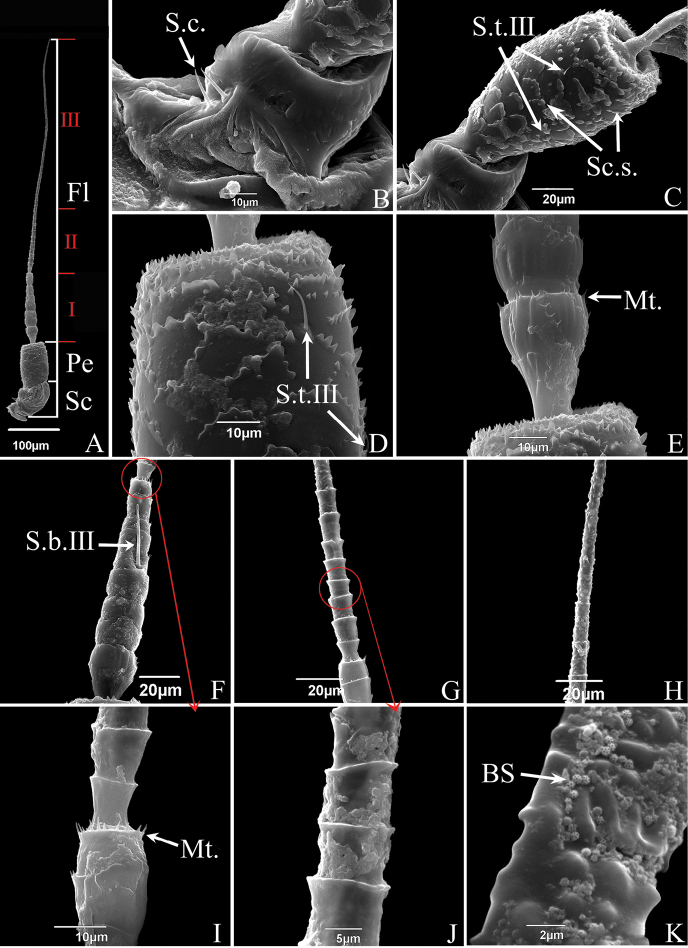
SEM of the antennae of *S.shinshana***A** antenna, composed of three parts: scape (Sc), pedicel (Pe) and three regions of flagellum (Fl) **B** scape, showing smooth surface, with two sensilla chaetica (S.c.) **C** pedicel, showing scale-like structures (Sc.s.) and sensilla trichodea III (S.t.III) **D** enlarged view of pedicel, showing scaly structures and sensilla trichodea III (S.t.III) **E** junction between pedicel and flagellum, showing microtrichia (Mt.) **F** first region of flagellum, showing sensilla basiconica III (S.b.III) **G** second region of the flagellum **H** junction between second and third regions of flagellum, showing change in surface protrusions **I** junction between first part and second regions of flagellum, showing microtrichia (Mt.) **J** enlarged view of second part of flagellum, showing cylindrical subsegments **K** enlarged view of third part of flagellum, showing brochosomes (BS).

**Figure 7.**
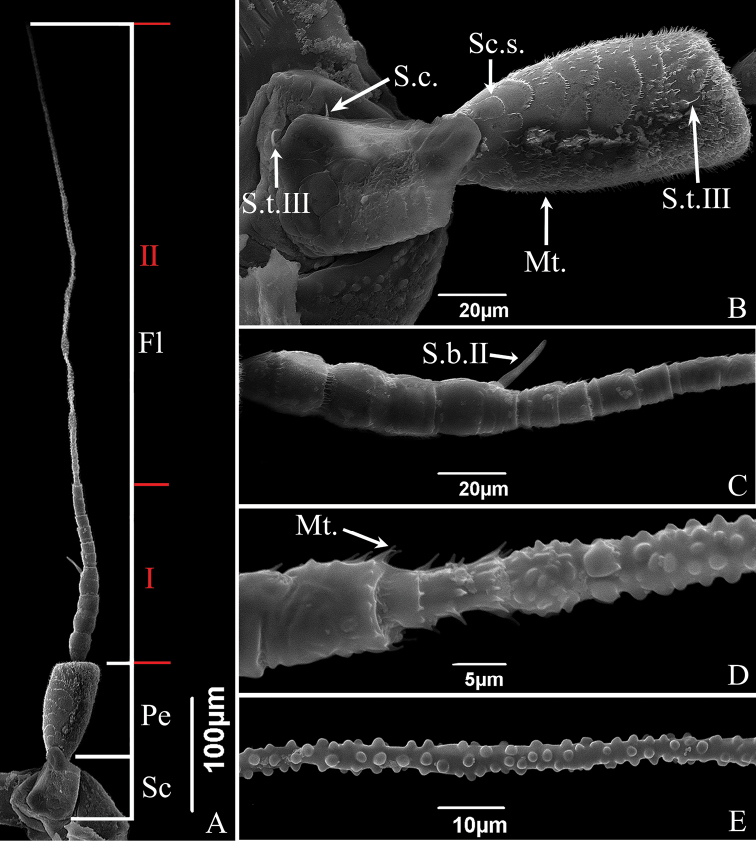
SEM of the antennae of *E.sipra***A** antenna, composed of three parts: scape (Sc), pedicel (Pe) and three regions of flagellum (Fl) **B** scape and pedicel, showing scale-like structures (Sc.s.), sensilla trichodea III (S.t.III), sensilla chaetica (S.c.), microtrichia (Mt.) **C** first region of flagellum, showing sensilla basiconica II (S.b.II) **D** junction between first and second regions of flagellum, showing change in surface protrusions and microtrichia (Mt.) **E** second region of the flagellum, showing spherical protrusions on the surface.

The antennae of the two studied species are of the typical arisoid type present in other Cicadellidae, composed of three parts: scape (Sc), pedicel (Pe) and flagellum (Fl) (Figs 6A, 7A). Their length relationship is: Sc < Pe < Fl, the flagellum is three times as long as the combined length of scape and pedicel. Measurements of each part of the antennae are summarized in Table 3. Except for the differences in length, there are no obvious differences in the morphology of the antennae of male and female adults and the distribution of sensilla. The distribution and numbers of sensilla are summarized in Table 4.

**Table 3.** Measurements of antennae (mean ± SE) obtained from scanning electron microscopy, *n* = 5. Sc: scape; Pe: pedicel; Fl: flagellum.

Segment		Sc		Pe		Fl		total length	
		*S.shinshana*	*E.sipra*	*S.shinshana*	*E.sipra*	*S.shinshana*	*E.sipra*	*S.shinshana*	*E.sipra*
Length (μm)	Male	58.7±3.9	52.7±9.6	78.3±6.8	72.9±6.1	518.5±14.1	496.9±13.5	655.5±25.9	622.5±29.2
	Female	59.1±2.8	56.4±8.1	80.6±9.2	75.6±10.4	548.7±25.1	513.3±12.3	688.4±37.1	645.3±30.8

**Table 4.** A statistical table of the sensilla and cuticular processes of the labium, antennae, and forewings. Lb-1: first segment of labium; Lb-1: second segment of labium; Lb-1: third segment of labium; Sc: scape; Pe: pedicel; Fl: flagellum; Fw: forewing. Note: The number of sensilla or cuticular processes is the average for the number of samples (*n* = 10); no entry indicates that the number of some sensors was not counted.

Sensilla type	Distribution (number)	
	*S.shinshana*	*E.sipra*
S.t. I	Lb-2(12); Lb-3	Lb-2(10); Lb-3(2)
S.t. II	Lb-2(4); Lb-3(4)	Lb-1(2); Lb-2(10); Lb-3
S.t. III	Lb-3(2); Pe(4)	Lb-3; Sc(1); Pe(2)
S.c.	Lb-3(2); Sc(2); Fw	Sc(1); Lb-3(2); Fw
S.b. I		Lb-1(2); Lb-3(2)
S.b. II	Lb-1(1); Lb-3(2)	Fl(1)
S.b. III	Fl(1)	
Pg.s.u. I	Lb-3(2)	
Pg.s.u. II		Lb-3(2)
S.co.	Lb-1(2)	
Sc.s.	Pe	Sc; Pe
C.p.	Lb-1	
Mt.	Lb-2; Lb-3; Fl; Fw	Lb-1; Lb-2; Lb-3; Pe; Fl; Fw

The scape is short, thick, approximately bell-shaped, with the base consisting of a flexible antennal membrane (Figs 6B, 7B). The scape of *E.sipra* has scalelike structures and microtrichia on the surface, while the scape of *S.shinshana* is relatively smooth without obvious surface sculpturing; the base in *E.sipra* has one sensilla chaetica and one sensilla trichodea III that are widely spaced, while the base in *S.shinshana* has two close-set sensilla chaetica (Figs 6B, 7B).

The pedicel is connected to the recessed socket at the end of the scape (Figs 6C, 7B). It is cylindrical, with many scale-like structures on the surface that gradually become fragmented from base to apex. The pedicel of *S.shinshana* has four sensilla trichodea III scattered on the surface, and the pedicel of *E.sipra* has two sensilla trichodea III and a large number of microtrichia (Figs 6C, D, 7B).

The flagellum is elongated and divided into numerous subsegments (Figs 6A, 7A). The flagellum of *S.shinshana* is divided into three morphologically distinct regions, while the flagellum of *E.sipra* is divided into two regions. The first (basal) region of *S.shinshana* is relatively thick and tapered, comprising the first nine subsegments, each with a large number of microtrichia (Fig. 6E, F, G, I). The first two subsegments are approximately bell-shaped and slightly swollen and widest distally, but the remaining subsegments of this section are more or less parallel sided (Fig. 6E, 6F). The second region starts from the tenth subsegment which is obviously narrowed compared to the previous subsegment; this region includes ten subsegments, each of which is cylindrical with no microtrichia at the apex but with the apex slightly flared (Fig. 6G–J). As in the first region, the first two subsegments of the second region are gradually expanded (Fig. 6G). The third region lacks has the subsegments more elongated and less well delimited, and has many protrusions of different sizes on the surface, giving the surface a rough, uneven appearance (Fig. 6H). From base to apex these protrusions gradually decrease in density; they are nearly spherical near the base and ridgelike near the apex (Fig. 6K). The first (basal) region of *E.sipra* consists of the eleven subsegments, with morphological characteristics similar to those of the basal region *S.shinshana* (Fig. 7A, C). The junction between the first region and the second region is significantly narrowed, and the second region lacks any indication of sub-segmentation, with many spherical protrusions on the surface but without ridgelike protrusions (Fig. 7D, E). The flagellum has only one long sensilla basiconica III (~ 40.67 μm in length) near the middle of the basal region (Fig. 6F). Both leafhoppers have a sensilla basiconica on their flagellum respectively (*S.shinshana*: sensilla basiconica III; *E.sipra*: sensilla basiconica II) near the middle of the basal region (Figs 6F, 7C).

**Figure 8.**
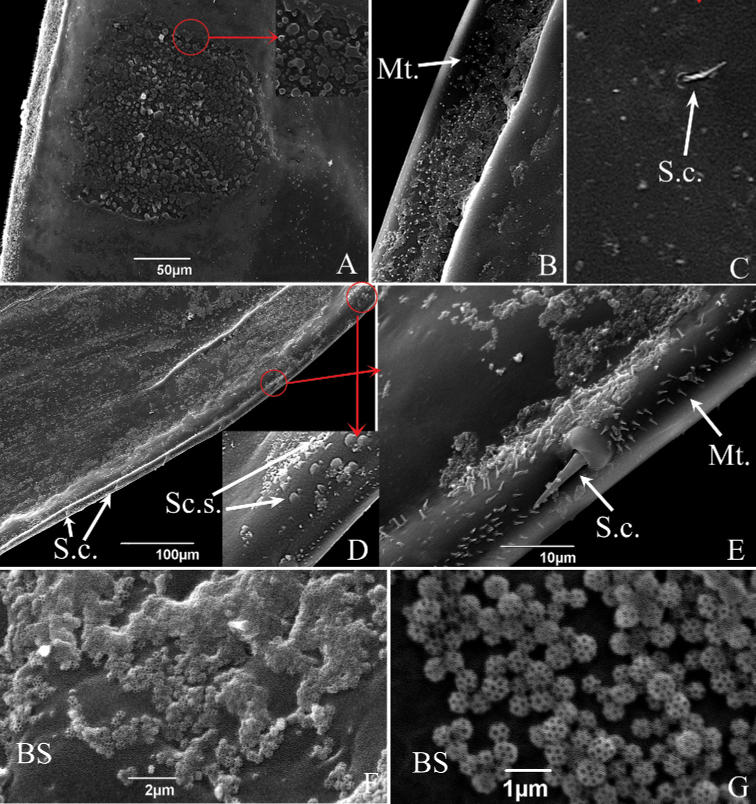
SEM of the brochosomes of *S.shinshana***A** peculiar fine structure of brochosomal area **B** microtrichia (Mt.) on transparent membrane of brochosomal area **C** sensilla chaetica (S.c.) on front edge of forewing **D** posterior edge of forewing, showing surface folds, scaly structure (Sc.s.) and sensilla chaetica (S.c.) **E** posterior edge of forewing, showing sensilla chaetica (S.c.) and microtrichia (Mt.) **F** brochosomes (BS) on front edge of forewing **G** enlarged view of brochosomes (BS) on front edge of forewing.

**Figure 9.**
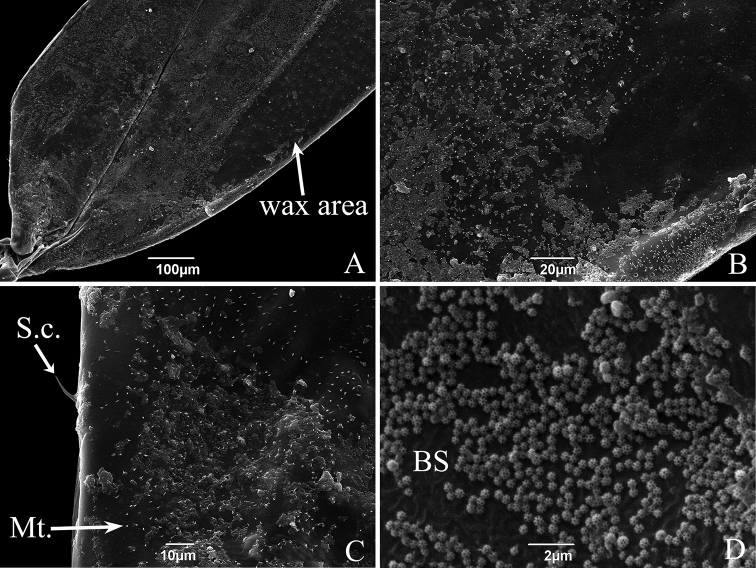
SEM of the brochosomes of *E.sipra***A** forewing, showing the distribution of brochosomes **B** the enlarged forewing part shows brochosomes and microtrichia (Mt.) **C** posterior edge of forewing, showing sensilla chaetica (S.c.) and microtrichia (Mt.) **D** brochosomes (BS).

As in other Typhlocybinae, the costal area has an elongated oval white area often referred to in previous literature as the “brochosomal area” or “wax field”, but actually consisting of a patch of brochosomes. There are numerous microtrichia and small sensilla chaetica scattered on upper forewing surface of the two erythroneurine species (Figs 8B–E, 9B, C); with a large number of microtrichia densely distributed on the transparent membrane of the front edge (costal margin) and a protruding ridge on the underside of the forewing (Figs 8A, B, 9B); relatively large sensilla chaetica are widely spaced along the edge of the forewing (Figs 8D, E, 9C). In addition, the forewing of *S.shinshana* has some small scalelike structures scattered around the hind edge (anal margin) near the base (Fig. 8D). Some samples have a unique microstructure near the forewing tip, which is composed of numerous rounded protrusions of various sizes and irregular shapes (Fig. 8A), and a few samples did not have this structure. No obvious differences in forewing fine structure were noted between male and female adults.

Small spherical brochosomes (the white powder on the forewings) were found on body surfaces of both male and female adults, with diameters of 402.00–583.10 nm (Figs 8G, 9D). Each brochosome is composed of multiple regular pentagonal and hexagonal cells partitioned by walls, the number of cells depends on the size of the brochosomes; smaller brochosomes have significantly fewer cells (Figs 8E, G, 9D). The brochosomes of *S.shinshana* and *E.sipra* are mostly concentrated at the base of the forewing, but there are fewer brochosomes on the brochosomal area (Figs 8A–D, 9A, B). The distribution of brochosomes on various surfaces of the body probably depends on how recently that individual leafhopper anointed and groomed itself with brochosomes. Observed under a scanning electron microscope, brochosomes are widely distributed on body surfaces of *S.shinshana* and *E.sipra*, with the largest concentrations usually on the hind legs, which are used by the leafhoppers during grooming to spread brochosomes over other parts of the body. When dense, brochosomes tend to gather together to form clumps (Fig. 8E). On mouthparts, brochosomes are mostly distributed on both sides of the longitudinal groove of the labium and around some sensilla (Figs 3A, B, D–I, 5H–M); on antennae, brochosomes are mostly distributed in the recesses of folds, and such distribution is most obvious in the distal region of the flagellum (Figs 6, 7).

## ﻿Discussion

Despite belonging to a single leafhopper tribe, the two studied species of Erythroneurini show remarkable differences in the fine structure of their mouthparts and antennae. The mouthparts of *S.shinshana* and *E.sipra* are generally similar to those of other Hemiptera in gross morphology (Tavella and Arzone 1993; Boyd 2003; Leopold et al. 2003; Wiesenborn 2004; Anderson et al. 2006; Zhao et al. 2010; Dai et al. 2014; Ge et al. 2016; Hao et al. 2016a, 2016b), but differ in many details including the fine structure of the labium and labrum, and the dentition of the stylets. Unlike most previously studied Hemiptera, which have protrusions on the labrum surface, some including sensilla (Leopold et al. 2003; Zhao et al. 2010; Dai et al. 2014; Hao et al. 2016a, 2016b), *S.shinshana* and *E.sipra* have the labrum surface with no sensilla. The labrum is similar to that of some aphids, e.g., *Eriosomalanigerum* (Hausmann, 1802) and *Aphiscitricola* Van der Goot, 1912, which have few labrum folds (Razaq et al. 2000; Ge et al, 2016). A few other studied leafhoppers, e.g., *Exitianusindicus* (Distant, 1908), *Laburrusimpictifrons* (Boheman, 1852) and *Aguriahanatriangularis* (Matsumura 1932) also have a smooth labrum (Pan 2013). This structure has been largely neglected in taxonomy and phylogenetic studies, but further comparative study of the labrum may show that its traits are useful for inferring relationships and distinguishing taxa.

The number of labium segments of Hemiptera insects varies between 1–5, but most species have 3 or 4 (Emeljanov 1987). Most Auchenorrhyncha have a three-segmented labium (*Lycormadelicatula* (White, 1845), with 5 segments, is an exception). The relative length of the segments can vary among species. The first segment of the labium of both *S.shinshana* and *E.sipra* are slightly shorter than the second. Although the cuticular processes on the first labium segment of the two species are different, such structures are common among leafhoppers (Zhao et al. 2010; Pan 2013; Hao et al. 2016b). Multiple sensilla are asymmetrically distributed along the longitudinal groove of the labium. Other sensilla present belong to more common types. We observed no clustered peg-structures on the tip of the labium as found in many other Auchenorrhyncha, only a pair of peg sensilla and a few sensilla trichodea are scattered on the surface, which is also seen in *Homalodiscavitripennis* (Germar, 1821), *Psammotettixstriatus* (Linnaeus, 1758), *Taurotettixelegans* (Melichar, 1900) and other leafhoppers (Leopold et al.2003; Zhao et al. 2010; Pan 2013). The structures at the tip of the labium are used to perceive the host plant surface. Some may also be used to rid the stylet fascicle of plant and salivary sheath debris during withdrawal of stylets from the plant tissue (Leopold et al. 2003). The specific roles of the various structures remain to be verified by further experiments.

The stylet fascicle is the main tool used for feeding, and it is also an important medium for spreading plant pathogens. *Singaporashinshana* and *E.sipra* have a ridge at the apex of the feeding stylet with a serrated structure in the middle. The ridges are not connected to the serrated structure, and their shape is very similar to that of *A.triangularis* (Pan 2013). Serrated structures were also found in other Hemipteran insects (Boyd 2003; Leopold et al. 2003; Anderson et al. 2006), but the numbers and shapes of teeth varies among species. These teeth cut channels into the plant tissues and help anchor the stylets during feeding. As in other Hemiptera, the interlocking part of the maxillary stylets of the two leafhoppers have a blunt and small toothed structure that facilitates tight coupling of the stylets during feeding. This is considered by Leopold et al. (2003) to be a ratchet device for positioning the stylets in apposition to each other.

Insect antennae are variously used in insect communication, foraging for food and courtship. Leafhopper antennae are relatively simple in structure and have relatively few sensory structures compared to those of some other Auchenorrhyncha (particularly Fulgoroidea); thus they have been little studies from a comparative perspective. The antennae of *S.shinshana* and *E.sipra* generally resemble those of other leafhoppers (Aljunid and Anderson 1983; Liang and Fletcher 2002; Romani et al. 2009; Stacconi and Romani 2012; Guo et al. 2018; Wang et al. 2018) but differ somewhat in fine structure. The scape of most previously studied leafhoppers has scale-like protrusions, as found in *E.sipra*, but the scape of *S.shinshana* has no protrusions and only has some shallow folds, which are similar to those found on the antennae of the lace bug (Tingidae) species *Stephanitisnashi* Esaki & Takeya, 1931 (Wang et al. 2020). This kind of scape is not common in Hemiptera, which usually have many projections on the surface, such as the papilla-like protrusions in *Sogatellafurcifera* (Horváth, 1899) in Delphacidae (Zhang et al. 2016), or reticular protrusions in *Triatomaguazu* Lent & Wygodzinsky, 1979 and *T.jurbergi* Carcavallo, Galvão & Lent, 1998 in Reduviidae (Silva et al. 2002).

The cylindrical pedicel is slightly longer than the scape. *Singaporashinshana* and *E.sipra* have scaly structures of different sizes scattered on the surface of the pedicel, but the cuticular processes that make up the scaly structure are different. The cuticular processes of *S.shinshana* are obviously wider than those of *E.sipra*. The scaly structure of *E.sipra* composed of micro-thorn-like cuticular processes is different from that of other leafhoppers (Mazzoni et al. 2009; Wang et al. 2018). This kind of microsculpture is similar to that found on the labium of this species and may represent the more generally distributed microsculpture pattern present on other external surfaces of this species.

The flagellum is the longest segment and has a large number of microtrichia at the end of each basal subsegment. Both *S.shinshana* and *E.sipra* have only one very long sensilla basiconica that appears on the 5^th^ subsegment of the flagellum. Previously studied leafhopper species, such as *Scaphoideustitanus* Ball, 1932, *Empoascaonukii* Matsuda, 1952, and *Chlorotettixnigromaculatus* (Dai, Chen & Li, 2006), have a longer sensillum between the 3^rd^ and 6^th^ subsegments of the flagellum (Mazzoni et al. 2009; Qiao et al. 2016; Guo et al. 2018). Although the antenna of leafhoppers remains little studied, perhaps because it does not appear to vary obviously among species when observed under light microscopy, the flagellum may be quite variable in fines structure among different leafhoppers. These differences are mainly manifested in the different numbers of segments, differences in the size and shape of the few sensilla present, and differences in the shapes of surface protrusions. For example, the flagella of *S.titanus* and *C.nigromaculatus* are sub-segmented from base to apex (Mazzoni et al. 2009; Guo et al. 2018), while the flagellum of *E.onukii* has only seven subsegments near the base (Qiao et al. 2016). The flagella of *S.shinshana* and *E.sipra* have numerous irregular protrusions but these differ in structure and density. Further comparative studies are needed to elucidate the morphological differences of these protrusions between species and their possible functions.

Brochosomes are minute protein-lipid particles with a net-like surface produced intracellularly in specialized glandular segments of the Malpighian tubules of leafhoppers. Their protein content ranges from 45–70% (Rakitov 2009; Rakitov et al. 2018). According to the shape, they are divided into two different types: integumental brochosomes (IBS) and egg brochosomes (EBS) by Rakitov (2009). The latter apparently occur only in some species of Proconiini in which the females exhibit a unique “egg-powdering” behavior. Rakitov (2004) also found that females of the genus *Proconia* are covered with a coating composed of large and small brochosomes, while the brochosomes of males are uniform in size and different from those of the female. The brochosomes of *S.shinshana* and *E.sipra* all appear to be the spherical type, similar to those found in other leafhoppers (Rakitov 1999, 2000, 2009; Humphrey and Dworakowska 2002). No differences in brochosome structure were observed between males and females.

After leafhoppers molt, brochosomes are secreted and anointed onto the body surface. Leafhopper species may differ in the amount of brochosomes secreted and in the time spent anointing. *Singaporashinshana* secretes 19 drops during each anointing episode on average, and the anointing behavior takes 2–4 h (Dong and Huang 2013). After leafhoppers secrete the liquid containing brochosomes, the liquid dries and gives rise to a visible pellet on the long oval “wax-area” of the front edge of the forewing. The fine structure of this area shows obvious differences among different species (Rakitov 1999, 2000). In order to improve adherence of brochosomes, the brochosomal area has horizontal ridges on the surface.

Brochosomes form a hydrophobic coating of the integument that can protect leafhoppers from wetting in areas of high humidity or rainfall. The brochosome coating may also provide some protection against high temperature and solar radiation, may help prevent evaporation of body surface water, and may also help leafhoppers avoid natural enemies, diseases, and parasites (Humphrey and Dworakowska 2002; Rakitov and Carolina 2005; Dong and Huang 2013), but most of these additional proposed benefits have yet to be proven.

## ﻿Conclusions

SEM comparisons of the integumental fine structure of two species of erythroneurine leafhoppers representing two different genera show that, although the overall structure of the mouthparts, antennae, and forewings are highly similar, many details differ between these species in integumental sculpturing, and the numbers, types, and distribution of sensilla. *Singaporashinshana* feeds on the leaves of peach and related Rosaceous trees while *E.sipra* and other species of *Empoascanara* feed on grasses. Thus, some of the observed differences may reflect adaptation to the very different chemical composition and structure of the host plants of these species. Further studies of other species in this tribe are needed to determine whether particular aspects of the mouthpart and antennal structures may be more broadly correlated to particular feeding preferences.
